# Improving Household Safety via a Dynamic Air Terminal Device in Order to Decrease Carbon Monoxide Migration from a Gas Furnace

**DOI:** 10.3390/ijerph19031676

**Published:** 2022-02-01

**Authors:** Nina Szczepanik-Scislo

**Affiliations:** Faculty of Environmental and Power Engineering, Cracow University of Technology, 31-155 Cracow, Poland; nszczepanik@pk.edu.pl

**Keywords:** CFD simulation, multizone simulation, CONTAM, carbon monoxide, air terminal device

## Abstract

The airtightness of buildings is continuing to grow and impact the indoor environment. Its aim is to conserve energy, but this may influence the indoor air quality and increase contaminant accumulation by limiting the amount of fresh air that infiltrates the building. The goal of this study was to quantify how the contaminants from a faulty gas furnace in a household could impact the occupants. The gas furnace was located in an attached garage and leaked carbon monoxide (CO). Multizone and CFD simulations were caried out to determine if an air terminal device (ATD) with a changing geometry could improve the air quality. The goal of the ATD was to maintain a steady air throw in the garage, while the air flow in the ventilation system would change. A steady air throw should help to remove the carbon monoxide generated from the furnace and prevent infiltration into the household. The results show that with the use of the new ATD, it was possible to maintain a steady air throw and the infiltration of CO was lowered.

## 1. Introduction

The growing airtightness of buildings is one of the main contributors of contaminant accumulation and migration within structures and it can pose a threat to the occupants’ wellbeing [1,2]. To combat this, newer ventilation strategies are being introduced to prevent occupants from harm and to provide good living conditions [3,4].

In residential households in Poland, it is popular for the garage to be attached to the house structure for the comfort of use. However, such an attached garage can contribute significantly to the indoor environment [5,6,7,8,9,10,11,12,13]. The contaminants from a starting car or chemicals stored in the garage can influence the air quality within an attached household. Additionally, in many homes the primary heating source is also located in the garage. If such a source, for example, a gas furnace, was to malfunction and the fume exhaust would be blocked, there is a risk of CO (carbon monoxide) poisoning. Due to its characteristics, carbon monoxide is extremely dangerous for humans. It is an odorless, tasteless, poisonous byproduct of partial combustion of fossil fuels that is a leading cause of brain injury and death [14,15]. Its high mortality is associated with impaired functioning of the red blood cells, affecting their ability to transport oxygen to the tissues in the body [16,17,18].

A previous study showed that accumulation of CO in a garage also elevated the levels of CO inside an attached household and that ventilation strategies have a large effect on the contaminant migration [19]. However, in this previous study, only natural ventilation was used. Because a growing number of residential houses are equipped with some form of mechanical ventilation system, it was decided to continue the study of the influence of ventilation elements and systems on improving the removal of contaminants.

While using mechanical ventilation or hybrid systems, the air supply is often connected to the heating and cooling systems. The amount of air that is supplied to a room is often controlled by the heat gains or losses in that room. Such control systems maintain proper thermal comfort for occupants inside a zone, so that the occupants do not overheat or overcool. Usually, the air throw lowers when using classical air terminal devices and the air flow lowers, leaving potential areas without fresh air. This can lead to the creation of dead zones, which could be dangerous for the occupants [20].

In this study, an air terminal device (ATD), whose role was to maintain a steady air throw, was examined. The air would reach the contaminant source despite the changes in the air flow and should remove the pollutants more efficiently.

To examine how the setting of the ATD influenced the CO concentration, a series of multizone and computational fluid dynamics (CFD) simulations were carried out using CONTAM with the CFD0 application.

CONTAM is a multizone computer program aimed at conducting an analysis of indoor air quality and ventilation [21]. It was designed by the National Institute of Standards and Technology (NIST) of the U.S. Department of Commerce and is used to assess air flows, personal exposure, contaminant concentrations, and contaminant migration. It has the ability to calculate building airflows and relative pressures between zones as well as determine the variation in ventilation rates for determining the distribution of ventilation air within a building [21]. It can be used to determine the indoor air quality (IAQ) performance of buildings before they are constructed and/or occupied and to investigate the impacts of different design decisions/methods concerning ventilation systems [22].

The program has been applied for multiple analyses, including contaminant transport [23,24,25,26,27] and infiltration calculations [28,29,30,31], and to monitor and analyze occupant exposure and comfort [4]. It is a valid tool in evaluating ventilation strategies and their influence on the indoor air quality (IAQ). However, the limitation of CONTAM is that it is a multizone program, meaning that each defined zone has a uniform contaminant concentration [21].

CFD simulation programs, on the other hand, make it possible to calculate detailed airflow patterns and thermal changes in various heating, ventilation, and air conditioning (HVAC) systems. They are often used for the estimation of the operation conditions of ventilation systems and their influence on occupants [20,32,33,34]. Additionally, they have been used to analyze the influence of structures on airflow [35,36,37,38] and contaminant transport [25,39,40]. CFD are usually limited to a closed zone, and it is not possible to provide results for an entire structure, such as a household.

Both multizone and CFD programs are widely used in research concerning IAQ and ventilation methods, which is why they were applied in this study. The combination of CONTAM with the CFD capabilities of the CFD0 were used to estimate the contaminant infiltration of CO from a faulty gas furnace in a garage into an attached house while using an adaptive ATD.

## 2. Materials and Methods

The aim of the research was to analyze whether an air terminal device with an adaptive geometry could improve the extraction of carbon monoxide in the case where a gas furnace had a blocked outlet vent, while simultaneously lowering the supplied fresh air. To achieve this, the CONTAM and CFD0 software created by the NIST were used.

When the outlet vent of a gas furnace is blocked, the most lethal contaminant that is produced is carbon monoxide. Because of its potential to quickly impact human health and even endanger human life, it was taken as a priority out of the contaminants that would occur in such a scenario. In a previous study, it was shown that a faulty exhaust line from the furnace not only resulted in a rise in CO in the room where the furnace was installed, but also in the adjoining building [19]. According to the World Health Organization (WHO), a person should not be subjected to a concentration of CO higher than 30 ppm (0.000055) in 1 h or 9 ppm (0.000017 kg/kg) during a period of 8 h [41]. The maximum amount of CO that a person should have contact with in a period of 15 min is 400 ppm (0.00072 kg/kg) as it is lethal after this time [42]

An ATD with adaptive geometry was analyzed to see if it would improve the removal of carbon monoxide in an attached garage of a residential house. The goal of the ATD was to maintain a steady air throw despite the changing conditions in the ventilation system when lowering the fresh air supply.

Because CONTAM is a multizone program, its limitations include the assumption that the pressure and contaminant concentration in each zone are uniform in each time step [43]. However, multizone modeling has the advantage, because it is able to simulate large objects with many different ventilation zones, allowing us to model infiltration as well as occupant exposure, while the occupant is active and moves in and out of the zones [24,44].

The air that flows from zone 1 to zone 2 in CONTAM is calculated based on the pressure drop between zones as is shown in Equation (1) [20]

(1) $$F_{1-2}=f\left(P_{1}-P_{2}\right)$$

where *F* is the flow (kg/s); and *P*_1_ and *P*_2_ are the pressures in the zones (Pa).

The mass of air in a zone is given by the ideal gas law

(2) $$m=\rho V=\frac{PV}{RT}$$

where *V* is the zone volume (m^3^); *P* is the pressure in the zone; *T* is the zone temperature (K); and *R* is the gas constant for air (–).

The contaminant analysis uses the same assumptions, and the air is treated as a mixture of several different contaminants. The inward airflow rate through one or more paths is calculated as:

(3) $$\sum_{2}F_{1-2}\left(1-\eta_{2}^{\alpha }\right)C_{2}^{\alpha }$$

where *F* is the mass flow rate between zones (kg/s) 1 and 2; $\eta_{2}^{\alpha }$ is the filter efficiency in the path; and $C_{2}^{\alpha }$ (kg/s) is the contaminant generation rate.

The above equations show that the program treats the airflow in a well-mixed way that influences the contaminant migration.

To analyze airflow and contaminant migration, the decision was made to combine CONTAM with the CFD0 editor that allows for a detailed CFD simulation of one zone.

**Figure 1:**
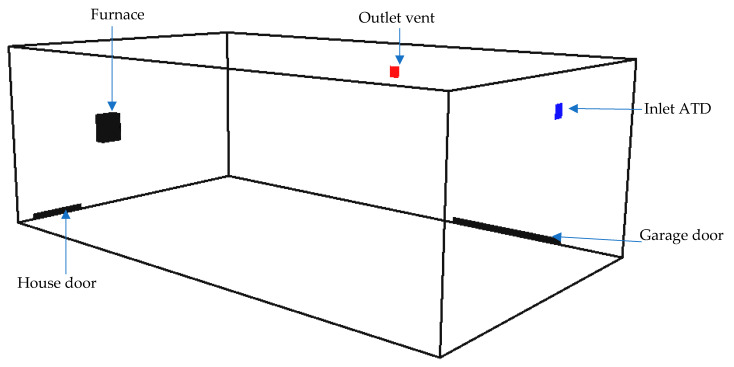
CFD0 wall frame of the garage.

The test zone was a garage with a faulty furnace, like in the previous study [19]. The chosen CFD zone was the garage and its layout on the CFD0 software can be seen in Figure 1. It had the dimensions of 7 m × 5 m × 3 m, to simulate a large garage. The outside temperature was assumed to be −10 °C to simulate the winter conditions, while the indoor temperature was assumed to be 20 °C. During the simulations, the energy equation option was used to consider the influence of the temperature difference of the outdoor air and the air inside the simulation zone. Contaminants were only generated in the garage to replicate the faulty furnace. The contaminant taken into consideration was carbon monoxide, as it is the deadliest out of the contaminants. The CO generation rate was assumed to be 1 × 10^−5^ kg/s, reflecting the average generation rate when there was a continuous operation of the furnace and the vent pipe was completely blocked [45]. The same generation rate and dimensions were used for the CONTAM as well as the CFD0 simulations. Because in the previous study only natural ventilation was taken into consideration [19], it was decided to continue the research and add an ATD connected to the mechanical ventilation system. The air flow would be lowered during the simulations.

The garage had multiple flow paths and openings. In the simulations, all except the inlet fan were power-law models, meaning that the airflow was calculated according to the differential pressure across the flow path and the flow coefficient [21]. The inlet fan was set as a mass-flow inlet that had a set flow. The same dimensions for the flow paths were used on the CFD0 simulations.

The garage was attached to a zone to resemble living quarters, the area of which was 60 m^2^, and an exchange rate of 1.5 1/h was used. The detailed layout is shown in [19].

The garage had the following flow paths attached to it:

- Garage door—0.03 m^2^;
- Door to the house—0.01 m^2^; and
- Inlet fan:
  ○ Max: 315 m^3^/h;
  ○ Med: 210 m^3^/h; and
  ○ Min: 150 m^3^/h.

CONTAM and CFD0 solve different sets of conservation equations. CONTAM only solves air mass and contaminant conservation equations, while CFD0 solves the Reynolds Averaged Navier–Stokes equations. The multizone model calculates average characteristics of airflow and contaminant transport, while the CFD method predicts three-dimensional distributions of the same parameters. Connecting CFD0 and CONTAM allows for detailed CFD zones in the CONTAM airflow and contaminant transport network.

The code of CFD0 is based on the finite volume method where the volume is divided into a set of small control cells, defined by a mesh. The mesh consisted of 2,000,000 elements and was consistent with the mesh in the previous study, where a mesh sensitivity study was caried out [19]. In this paper, the simulation was solved with the k-ε turbulence model and wall functions [46], which is one of the options alongside the laminar and zero-equation models in this software. The contaminant and airflow simulation methods were set to transient. Boundary conditions were imported from the CONTAM software, and a coupled simulation was conducted (CONTAM → CFD → CONTAM). In this coupling method, CONTAM provides a pressure boundary condition to CFD0. Later, the results from the zone are returned to CONTAM. This methodology was selected for this research as it provides a stable and convergent coupling solution [46].

Equation residuals were used to evaluate the convergence and solutions were considered converged when the scaled residuals were less than 10^−6^ for the momentum, turbulence dissipation, energy, and species [47,48].

The role of the installed ATD was to maintain a steady air throw, meaning the distance the fresh air reaches should be constant, despite the changing conditions. The assumed cut off air velocity that determined the air throw was 0.5 m/s. The ATD was tested in a laboratory environment and could maintain a steady throw of up to 7 m when using the same three airflows as were used in this study. The layout of the setup can be found in [49]. To determine if the CFD0 software could show the changing conditions, simulations were carried out for the medium flow. The velocity along the axis of the flow was both measured on the laboratory stand as well as calculated in the simulation. The ATD in the simulations had the same hydraulic diameter as in the laboratory stand. The comparison of the two tests can be found in Table 1. The convergence rate was around 11% with the experimental data, which is satisfactory for a simulation when using CFD0.

In this study, four different cases were taken into consideration:

- Case 1: the maximum 0.1024 kg/s (315 m^3^/h) flow was used and the maximum surface area of the ATD, Effective ATD area: 0.030961 m^2^;
- Case 2: the minimum flow 0.0417 kg/s (150 m^3^/h) was used and the maximum surface area of the ATD, Effective ATD area: 0.030961 m^2^;
- Case 3: the medium flow 0.0611 kg/s (220 m^3^/h) was used and the medium surface area of the ATD, Effective ATD area 0.019745 m^2^; and
- Case 4: the minimum flow 0.0417 kg/s (150 m^3^/h) was used and the minimum surface area of the ATD, Effective ATD area 0.007631 m^2^.

For all cases, both the multizone and CFD simulations were carried out. All input parameters were identical; the only changes were in the size of the inlet vent and the air flow magnitude. The time frame for each simulation was 12 h.

## 3. Results

### 3.1. Case 1: Maximum Flow and Maximum Diameter

**Figure 2:**
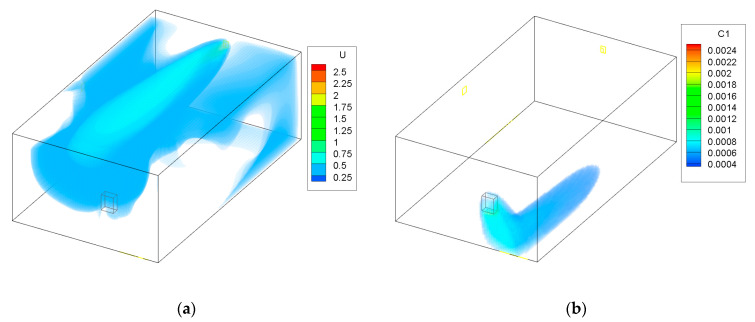
Case 1—maximum flow and maximum area of the ATD; (**a**) air velocity (m/s), (**b**) CO contraction (kg/kg).

The results for Case 1 are shown in Figure 2. In this case, the maximum diameter and air flow were used. Fresh air flows into the zone from the opposite end to the furnace. The fresh air reaches the furnace and dilutes and then removes the CO from the zone. The high concentration only remains near the contaminant source. Thanks to this, the spreading of CO is limited and CO is removed from the zone.

### 3.2. Case 2: Minimum Flow and Maximum Diameter

**Figure 3:**
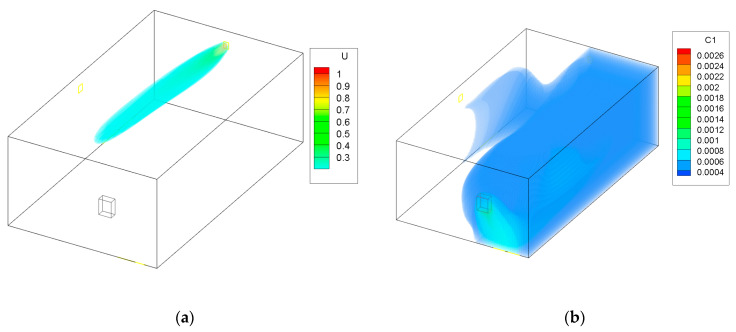
Case 2—minimum flow and maximum area of the ATD; (**a**) air velocity (m/s), (**b**) CO contraction (kg/kg).

To see how the contaminants would spread without the use of the adaptive ATD, a simulation was preformed using the largest cross section of the ATD and the minimal flow. The results are presented in Figure 3. It shows that the fresh air does not reach the gas burner and that the CO spread is wider throughout the garage. This can be harmful for occupants entering the garage that would risk exposure to large quantities of dangerous contaminants.

### 3.3. Case 3 and Case 4

**Figure 4:**
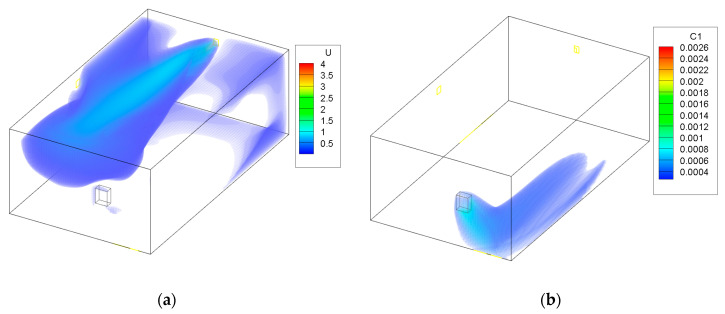
Case 3—medium flow and medium area of the ATD; (**a**) air velocity (m/s), (**b**) CO contraction (kg/kg).

**Figure 5:**
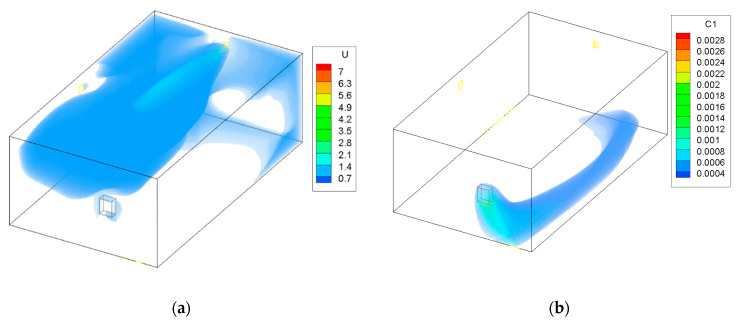
Case 4—minimum flow and minimum area of the ATD; (**a**) air velocity (m/s), (**b**) CO contraction (kg/kg).

The next cases involved the study when using the adaptive ATD while lowering the air flow through the system. The results are shown in Figure 4 and Figure 5. The figures show that despite lowering the air flow, fresh air is still provided to the gas furnace. The air throw is maintained and there is no threat of the formation of dead zones. The air throw in mixing ventilation systems, such as in this study, is a curtailing factor when considering contaminant removal. A good air throw allows the fresh air to reach each vital zone within a ventilated room. The use of the ATD allowed the air to be distributed in a large area using only one element of the ventilation system. A problem that might occur is that the air velocity near the ATD can increase when the cross section is smaller. This could lead to a lack of thermal comfort in the region closer to the device. However, it is applied in the garage, which is not a residential room of a household and occupants do not spend long periods of time in it.

The area of the garage that is without coloring in Figure 2, Figure 3, Figure 4 and Figure 5 has a concentration of CO below 0.0003 kg/kg; this was done to improve the clarity of the figures.

### 3.4. Contamiant Concentration

Adding the ATD to the garage allowed the fresh air to maintain a steady air throw and allowed the fresh air to reach the faulty gas burner, but did this contribute to the minimization of contaminant leakage into the attached household?

**Figure 6:**
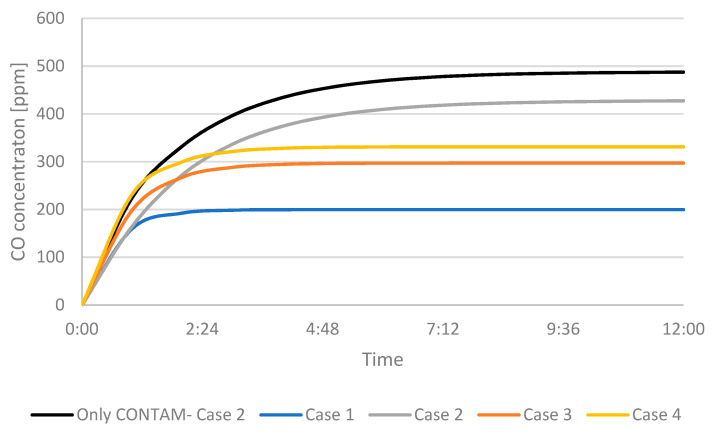
CO concentration in the garage.

**Figure 7:**
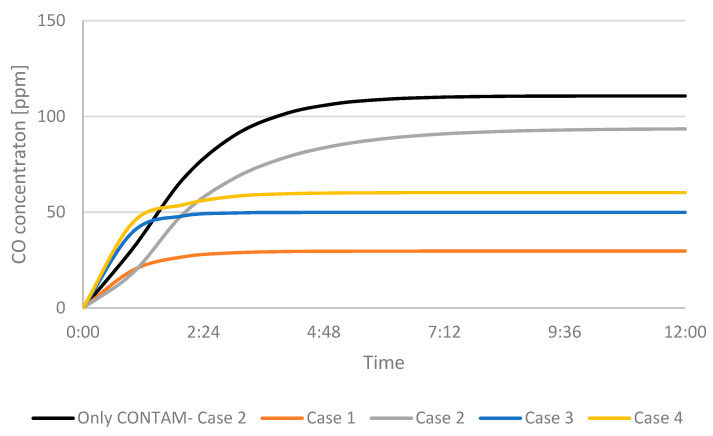
CO concentration in the adjacent room.

Figure 6 shows the CO concentration in the garage, while Figure 7 shows the concentration in the adjoining room that is connected to the garage through a door from the CONTAM software. The figures show the cases in which only the multizone program was used, as well as the results when the program was coupled with the CFD simulations. Because CONTAM assumes the concentration of contaminants to be uniform, it does not provide information about in what region of the garage the concentration of CO might be harmful for occupants as is presented in Figure 2, Figure 3, Figure 4 and Figure 5.

Figure 6 shows that the concentration inside of the garage itself with the furnace is above 200 ppm in each case, which can be life threatening. The highest concentration is for Case 2, where the airflow was lowered to a minimum without changing the effective area of the ATD. It lowers by almost 100 ppm when using the new ATD, proving that it can elevate the IAQ. The best-case scenario is when the maximum air flow was used.

Similar results are shown for the adjoining room in Figure 7. Like the results of the garage, the concentration of contaminants when only using the multizone program is higher than when considering the air flow layout by using the CFD application. Analogous results were observed in the previous study [19]. Again, in Figure 7 the effects of using the ATD are shown. The CO concentration is lower when using the device for Case 3 and 4 comparing with Case 2, where the device was not used. The fresh air was able to reach the faulty furnace and remove the contaminants more efficiently before they were transported to the adjoining room. Again, the best results were achieved when the maximum airflow was used.

The conducted simulations assumed that there was no CO sensor, which could warn the occupants. The results prove that they are a necessity, as such accumulation is extremely dangerous. It would warn the occupants much sooner and/or be integrated with the ventilation system, thus improving safety.

## 4. Conclusions

The aim of the study was to evaluate the effectiveness of an air terminal device with a changing geometry when concerning the improvement of air quality. The simulation program CONTAM and the CFD0 application were used. The goal of the ATD was to maintain a steady air throw in a large garage to remove carbon monoxide generated from a furnace with a blocked exhaust vent. The contaminant was leaking into the garage and the adjoining room through a leak space underneath a doorway. The air supply was lowered gradually.

The goal of the ATD was to maintain a steady air throw despite the lowering air flow to help improve contaminant removal. The effective area of the ATD was changed when the supply air volume was lowered to maintain a constant supply of fresh air to the contaminant source. The change in the geometry of the element allowed the air to reach the gas furnace despite the lowering of the air supply.

The results also show that without the ATD, the CO concentration in the adjoining zone was higher as fresh air did not reach the furnace. This could potentially be life threatening for occupants both inside the garage and the house it is attached to. When using the proposed ATD while lowering the air flow, the air still reached the gas furnace, allowing for the removal of more of the deadly contaminant. In effect, this could allow the occupants to have enough time to remove the source of CO before the situation became life threatening.

The thermal effects of using the ATD as the air supply were not studied in this paper. However, one of the main faults of this solution is the increased air speed near the ATD that would lower the thermal comfort for occupants and the expected cooling effect of the cold air. The garage itself is not a residential space of the house, and the comfort can be overlooked when considering the safety it may provide. However, further simulations will be conducted to combat the issue of thermal comfort and for further improvement of ventilation strategies.

## Figures and Tables

**Table 1:** Laboratory measurement and CFD simulation comparison of average air velocity.

Distance from ATD (m)	Average Air Velocity V (m/s)		Convergence Rate (%)
	Measured	Numerical	
0	4.05	4.40	9
2	1.98	1.77	11
5	0.76	0.67	12

## Data Availability

Not applicable.
